# Director Field Model of the Primary Visual Cortex for Contour Detection

**DOI:** 10.1371/journal.pone.0108991

**Published:** 2014-10-17

**Authors:** Vijay Singh, Martin Tchernookov, Rebecca Butterfield, Ilya Nemenman

**Affiliations:** 1 Department of Physics, Emory University, Atlanta, GA, United States of America; 2 Department of Biology, Emory University, Atlanta, GA, United States of America; Xiamen University, China

## Abstract

We aim to build the simplest possible model capable of detecting long, noisy contours in a cluttered visual scene. For this, we model the neural dynamics in the primate primary visual cortex in terms of a continuous director field that describes the average rate and the average orientational preference of active neurons at a particular point in the cortex. We then use a linear-nonlinear dynamical model with long range connectivity patterns to enforce long-range statistical context present in the analyzed images. The resulting model has substantially fewer degrees of freedom than traditional models, and yet it can distinguish large contiguous objects from the background clutter by suppressing the clutter and by filling-in occluded elements of object contours. This results in high-precision, high-recall detection of large objects in cluttered scenes. Parenthetically, our model has a direct correspondence with the Landau - de Gennes theory of nematic liquid crystal in two dimensions.

## Introduction

**Figure 1:**
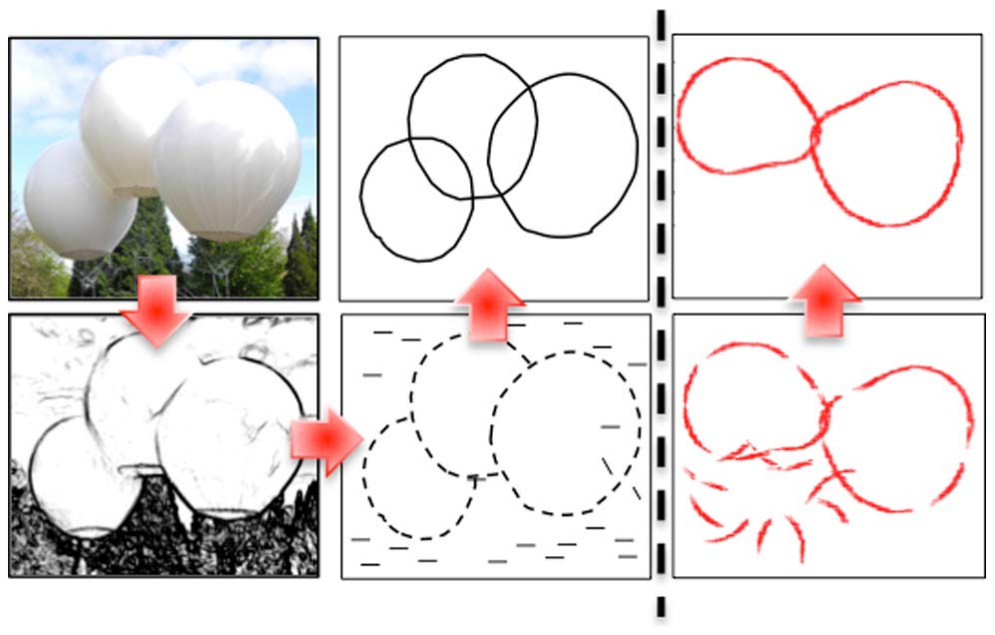
Contour Reconstruction Task. A 2d image (left top; credit: ‘Pont de Singe’, Olivier Grossetete. Photo: Thierry Bal) is recorded as a field of contrast by the retina and the LGN (left bottom). V1 neurons respond to regions of contrast changes in a direction-selective manner, performing edge detection (middle bottom). The information from edges is integrated to reconstruct long contours (middle top). In this paper, we model the visual process starting from edges in V1; sample input (bottom) and output (top) to our model are on the right.

To recognize an object in a visual scene, humans and other primates process visual signals relayed through the retina [1] in the ventral stream of the cortex. Contour detection is a crucial part of this process (Fig. 1). It is carried out at early stages of the processing in the primary visual cortex (V1) of the brain [2]. V1 consists of hundreds of millions of neurons organized topographically into columns of 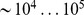 neurons each. Neurons in each column receive inputs from a localized part of the visual field (called classical, or feed-forward receptive field). They are directionally selective, responding primarily to oriented edges within their receptive fields [3], [4]. Computational vision models that account for such receptive fields of individual neurons [5]–[10] typically incorporate them within feedforward hierarchical structures similar to the cortex [11]–[13]. Such feedforward models account for the visual processes on short time scales, and achieve error rates as low as 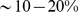 on typical object detection tasks [10], [14].

It is believed that, *in vivo*, the error rate is reduced by orders of magnitude by contextual information that influences local processing, which may not be captured fully in such models [15], [16]. These collective, recurrent dynamics span large spatiotemporal scales and are mediated through thousands of axons laterally connecting distant columns [17]. These interactions are believed to suppress the clutter present in the visual field, while simultaneously binding edges into contours [18].

The goal of this paper is to build *the simplest model of the primary visual cortex* that simultaneously achieves two contradictory tasks: clutter removal and occlusion filling. We do not aim at the state of the art performance on complex natural images, but rather ask what is the smallest set of computational primitives that must be implemented in a model to achieve such detection and integration of long contours in a nontrivial setting. For this, we focus on a proposal of a specific lateral connectivity among V1 neurons [19], [20], which incorporates the Hebbian constraint that neurons that are excited simultaneously by the same long, low-curvature contours should activate each other [19]. However, in our model, we do not reproduce the complexity of V1, which has 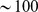 million neurons, with each neuron having 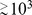 connections, some extending for many millimeters. Instead, unlike most agent based discrete models, we represent the activity as a *coarse-grained, continuous neural field*, which we model as a complex-valued field on the complex plane, 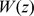. The magnitude and the phase of 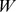 represents the level of excitation and the orientation of the dominant contour element at point 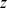, respectively. This coarse graining helps us to identify the minimal features of the neural structure and dynamics that are essential for contour recognition.

Importantly, our complex field approach is significantly simpler than most other coarse-grained models, thus pushing the limits in identification of the minimal set of the required computational primitives. Indeed, typically the neural firing rate is represented as a real function of three variables (position in the visual plane and the directional sensitivity) [21], [22]. In our model, the firing rate is represented as a complex function of a complex variable (or, equivalently, two real variables), which, manifestly, has a lot fewer degrees of freedom. Previous approaches that used a similar complex field representation [23], [24] have focused on development, rather than on the visual performance of the cortex. Thus it has been unclear if the simplified, lower-dimensional model can solve complex visual tasks. Here we answer this question affirmatively.

## Model

We define the dynamical variables in our coarse-grained model as the neural firing rate 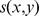, 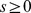, over the two dimensional plane 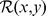, and the orientation preference 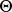 of neurons, both averaged over a microscopic patch of the cortex, which still contains many thousands of neurons. Such averaging is traditional in, for example, fluid dynamics, where continuous dynamics is sought from discrete agents. The neural activity is invariant under parity (i. e., an edge or its 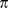 rotation results in the same activity). Further, two equal edges at one point oriented 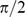 apart lead to cross orientation suppression, not forming a dominant orientation at the point [25]. Thus the fields 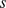 and 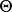 are combined into a time varying complex field 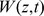 in a somewhat uncommon way, forming an object called a *director* [26]: 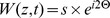. The magnitude of this field is the average firing rate, and the argument is twice the average dominant orientation preference of neurons at a point 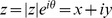 [23], [24]. We similarly coarse-grain the input images, identifying the dominant orientation at every point (see *Methods*). This orientation field serves as the input to the model. Note the crucial reduction in the number of degrees of freedom in going from a more traditional description 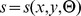 to 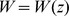. One of the costs of the simplification is the lost ability to represent multiple different orientations at the same point, which happens when contours intersect. Correspondingly, one of our goals is to verify that this loss does not make it impossible to perform non-trivial visual tasks.

**Figure 2:**
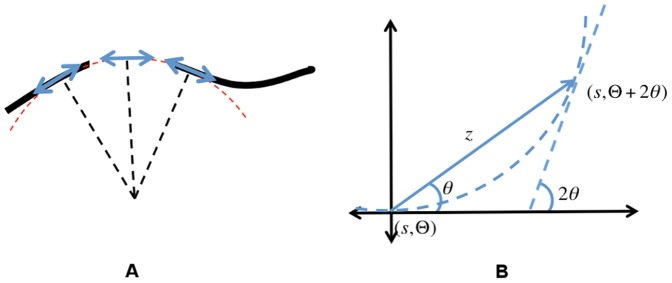
Co-circularity condition. (A) Neurons send excitatory signals along approximately co-circular directions. Thus neurons in occluded gaps may get enough excitatory input along smooth contours to get excited without direct visual input. (B) The orientation at two points is said to be co-circular if they are tangential to the circle connecting the two points. If the orientation preference at the origin is along the real axis, the co-circular edge at a point 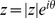 has the orientation 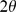. Multiplication by 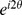 can be written as: 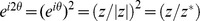.

Neurophysiological and psychophysical experiments [15], [16], [18], [27], [28] and theoretical considerations [19] suggest that neurons in V1 are laterally connected such that active neurons excite nearby neurons with collinear or large-radius co-circular directional preference. Conceptually, simultaneous input from several collinear or co-circular neurons can excite other neurons that might otherwise not be getting enough excitation from the visual field due to occlusion or noise, cf. Fig. 2 **A**. At the same time, neurons responding to high spatial frequency clutter elements do not get sufficient lateral excitation, and their activity decays. These collective dynamics integrate information over large spatial scales.

We represent these phenomena in a traditional linear-nonlinear model, where the neural field at a point 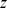 is affected by a combination of lateral synaptic inputs:
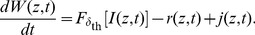
(1)


Here 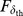 is some sigmoidal function of the excitatory input 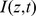, 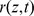 describes the inhibitory contribution to the field, and 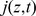 is the stimulus.

The excitatory input, 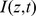, combines synaptic input from all points 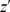 in its interaction region ‘Ex’
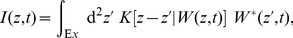
(2)where 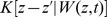 is the excitatory interaction kernel between the fields at point 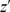 and 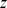, when the field at 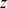 is 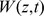. The kernel for an arbitrary orientation of 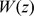 can be defined by an appropriate rotation of the kernel defined for 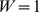 (parallel to the real axis):
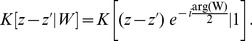
(3)


Co-circular excitation may be represented as
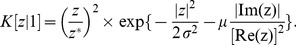
(4)


**Figure 3:**
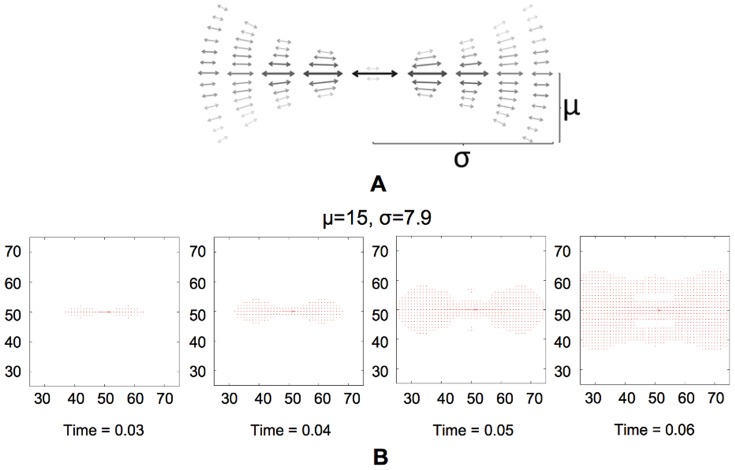
Shape of the interaction kernel. (A)Schematic shape of the interaction kernel 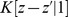. Arrows represent the orientation preference and darkness and size represent the magnitude. (B) Results of dynamics with the kernel 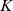 with the current 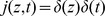. Here, as everywhere in this work, we use 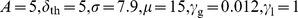, which optimizes the performance according to a genetic algorithm search over the parameter space, see *Methods*.

The first term, derived in Fig. 2, determines the field direction at 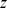 that is co-circular to the field at 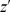. The 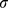 term in the exponent determines the spatial range of the excitation. The 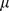 term determines the smallest radius for which substantial co-circular excitations still exist, giving the kernel and hence the induced dynamics their characteristic bow-tie shapes [19], see Fig. 3. Note again the reduced complexity of this model, where the kernel is defined by just two real-valued parameters, instead of being inferred empirically from the data in a form of a multi-dimensional matrix, as in Ref. [20] and references therein.

We define the input nonlinearity using a complex step function:
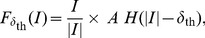
(5)where 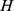 is the Heaviside step function and 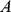 determines the maximum excitation strength. Smoother sigmoidal nonlinearities were tried as well, but this had little effect on the results presented below. If the total excitatory input is higher than the threshold 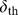, then the field 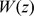 gets a positive increment in the direction of the total input. For this, the excitatory contribution from a large part of the neural field must align in the same direction, representing coincidence detection. While importance of this coincidence detection phenomenon in vision is unclear, it is crucial in the context of auditory signal processing [29]. Thresholding also suppresses clutter-induced spurious excitations, as it is unlikely that the excitatory input from short clutter elements becomes higher than the threshold in the absence of contextual support from long contours.

The inhibition term 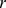 represents two distinct phenomena: local relaxation, which depends on the local field magnitude [30], and global inhibition [31], which keeps the activity of the entire neural field in check (presumably through intermediate inhibitory neurons, not modeled explicitly). In the spirit of writing the simplest possible model, we represent inhibition as linear, resulting in:
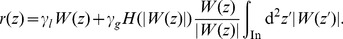
(6)


Here 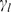 and 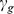 determine the rates of local and global inhibition, and ‘In’ stands for the range of global inhibitory interactions. Combined with the non-linear excitation, this linear inhibition produces bimodal asymptotic field values. Hence, neurons can be defined as ‘active’ or not.

## Methods

### Image generation

Since our focus is not on practical image processing algorithms, we focus on synthetic images in this work, as in [20]. This makes it easier to analyze effects of various image properties on the performance.

#### Targets

The “amoeba” objects (long closed contours with gaps) are generated by choosing a center at a random point in the image, and then drawing the amoeba around this point in polar coordinates, with the radius as a superposition of periodic functions with different radial frequencies, 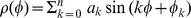. The Fourier coefficients 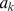 are generated randomly from a normal distribution (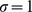), with 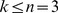, and the phases 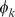 are uniformly distributed between 0 and 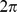. To create amoebas that are about the same size, the coefficients are further constrained such that the minimum and the maximum radii of the resulting amoeba and their ratios obey 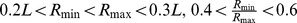, where 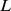 is the image size. The input current then is 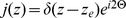, for every point 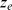 within 1 lattice spacing away from any point on the amoeba contour, where 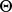 is tangential to the contour at that point. While generating an amoeba, we also determine an exclusion region around it of 8 lattice sites. Clutter elements (see below) with orientations parallel to the closest amoeba segment are not allowed in these regions. Without such exclusion, a nearby clutter edge could help amoeba detection, which would artificially elevate the measured performance. We prefer to err on the side of underestimating the performance, and hence we remove these ambiguous cases.

#### Occlusions

We simulate occlusions and noise in real-world images by removing parts of amoebas. A random number of 2–4 segments with random angular length combining to the total of 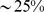 of the amoeba length are chosen at random positions along the amoeba contour. Within the chosen segments, the input current 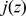 is then set to zero.

#### Clutter

We need the clutter to be indistinguishable from the targets by curvature, brightness, and other local statistics, so that object detection is impossible without long-range contextual contour integration afforded by co-circular connectivity. Thus clutter is generated by first generating an amoeba as described above, partitioning it into segments, and then randomly shuffling and rotating the segments to break long-range contour continuity. Specifically, the model cortex is divided into 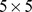 square regions, which are then randomly permuted. The center-of-mass (CoM) of an image within each region is computed, and the dominant angular orientation is determined. Then each region is rotated around its CoM by a random angle, subject to a constraint that the resulting dominant orientations of neighboring regions are different. The constraint ensures that the clutter does not form long range target-like structures.

#### Combined images

**Figure 4:**
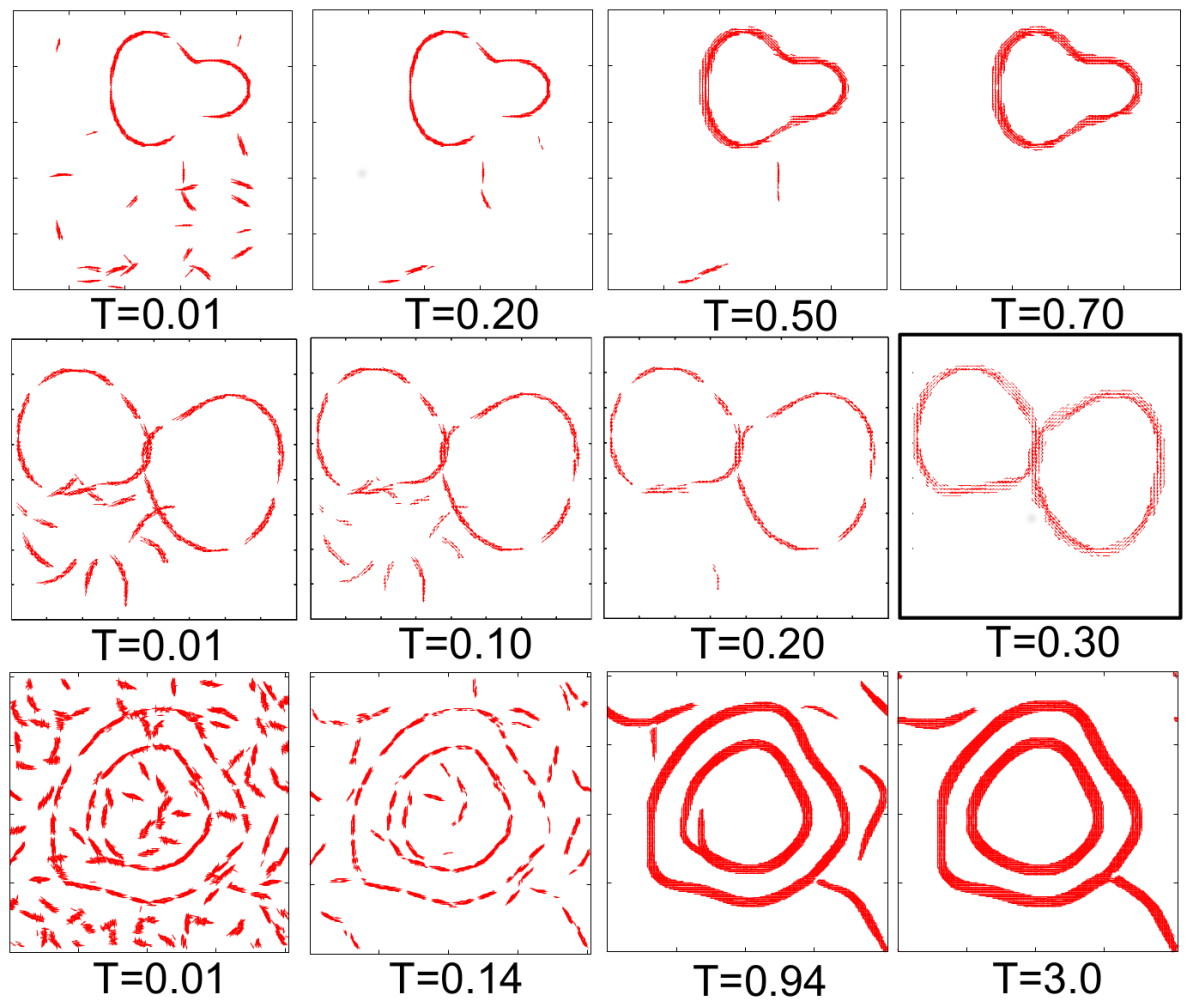
Neural field dynamics. (top and middle) Time evolution of the neural field for sample images. The magnitude (line width) and the direction of the field are plotted at every point where the strength of the field is higher than a cutoff (0.35). The parameters of the dynamics are as in Fig. 3. Dynamics removes the clutter and fills in the occlusion gaps. However, spurious activity (widening lines) appears for large simulation times, so that the best performance is obtained for intermediate times. (bottom) Performance of the model on an image used in psychophysics experiments [32]. Like human subjects, the model can identify, complete, and bind together long punctuated contours.

One or two targets and clutter resulting from breakup of one or two additional targets were then superimposed together to form test images, see Fig. 4, for an example. Clutter in the exclusion zones along the amoeba contours was then removed, as described above.

#### Transforming pixel images

Images used previously in psychophysics experiment (Fig. 4) were imported into MATLAB and then converted to grey scale using rgb2grey. The resulting matrix was then thresholded and converted into a binary matrix. A 2D Gabor filter was used to find edges in this bitmap image. For each point in the image, we find the convolution of a Gabor filter 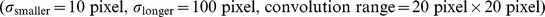 with the image at 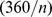 angles where 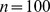. The direction with the maximum convolution is taken as the orientation of the visual field at the point, and the result of the convolution as the field magnitude. The image thus processed is presented as an input for simulations.

### Simulations

The time evolution of the model is studied on a square lattice of a linear size 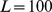 with periodic boundary conditions using Euler iteration method. The lattice discretization is done for simulation purposes, and should not be viewed as a representation of discrete neurons; we are not aware of numerical algorithms able to simulated our model dynamics without discretizing the space first.

In each iteration cycle we first calculate the total input 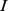 at each point 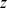 from all other points 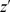 in the excitation region ‘Ex’ using a precomputed interaction kernel 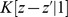 on a 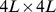 *kernel lattice*. Square discretization destroys the angular symmetry of the kernel evaluated at an arbitrary 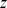. The following procedure restored the symmetry. First, to calculate the contribution from 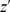 to 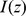, the kernel lattice is superimposed on the image lattice with the origin of the kernel lattice at point 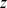 of the image lattice. Next the kernel lattice is rotated by 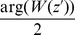 with respect to the image. Then the contribution from the point 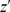 to 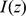 is 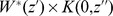, where 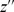 is the point on kernel lattice closest to 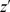. The total input 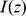 is then the sum of contributions from all points 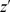 in the excitatory interaction region ‘Ex’. After the input is calculated, if 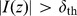, then the field is incremented 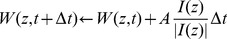, where 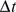 is the time step. To account for degradation, we finally set 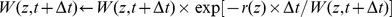, where 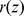 is as in Eq. (5). To the first order in 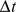, this is equivalent to the dynamics in Eq. (1). However, this exponential form removes the large fluctuations in 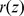 when 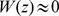.

In our simulations, the excitation range ‘Ex’ is 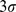, where 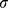 is the effective spatial range of the kernel 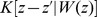. For global inhibition range ‘In’ is the entire lattice. The model is easily modified to restrict the suppression to a smaller inhibition region.

We first chose the parameter 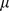 to be similar to the curvature of a typical amoeba. Next 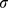 was chosen such that it was larger than the typical extent of the occluded amoeba segments. The initial values of 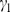 and 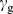 were determined using steady state analysis of the model, which leads to 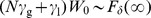, where 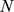 is the typical number of points with non zero field, and 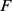 is the thresholding function as defined in Eq. (1). Setting 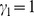 and 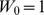, we thus constrain all other parameters. Using these initial values, some coarse parameter optimization was done by simply observing the simulations while the parameters were varied. After that genetic algorithm was used to optimize the model for maximum simultaneous precision and recall (see Results for definitions). We used the area under the precision-recall curve as our fitness function. Parameters were changed by a percentage drawn from a uniform distribution (from −1% to 1%) and the fitness function was recalculated for the new parameters. Then, the new parameters were either accepted or rejected according to whether 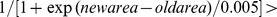 random variable drawn from uniform distribution on (0,1). The parameter 0.005 acts as the temperature. The final optimized values of the parameters used for simulations presented here were: 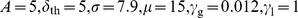.

The code was implemented in C, compiled with the gcc v. 4.7, and optimized with OpenMP libraries. Simulations were performed on a computer with Intel i7 2600k (clock speed 3.4 GHz). The simulation time for 250 iteration cycles for one image took about 10 s. All model dynamics times were measured in units of 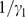, which was set to 1 in our simulations.

## Results

Figure 4 (top and middle) shows the time evolution of the neural field 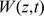 in our coarse-grained model for a sample input image, generated as described in *Methods*, where a large contiguous contour with gaps (an amoeba) is superimposed on clutter. The gaps model occlusion of contours by other objects and noise in the earlier stages of visual processing. Similarly, Figure 4 (bottom) illustrates the model output for an image previously used in psychophysics experiments with human subjects [32]. Its simplicity notwithstanding, the model performs qualitatively similar to humans in that long contours implied by collinearity of nearby edge segments are easily detected. The gaps in amoeba targets get filled, while the clutter decays with time, resulting in emergence of long contours. Note also that spurious activity appears around contours at large simulation times. Even though such hallucinations rarely happen in human vision, they are not of a big concern here since, at large times, the dynamics would be affected by feedback from higher cortical areas and eye movements, which we are not modeling. Importantly, these observation suggests that the model performance must be evaluated at finite, but not asymptotically large times.

**Figure 5:**
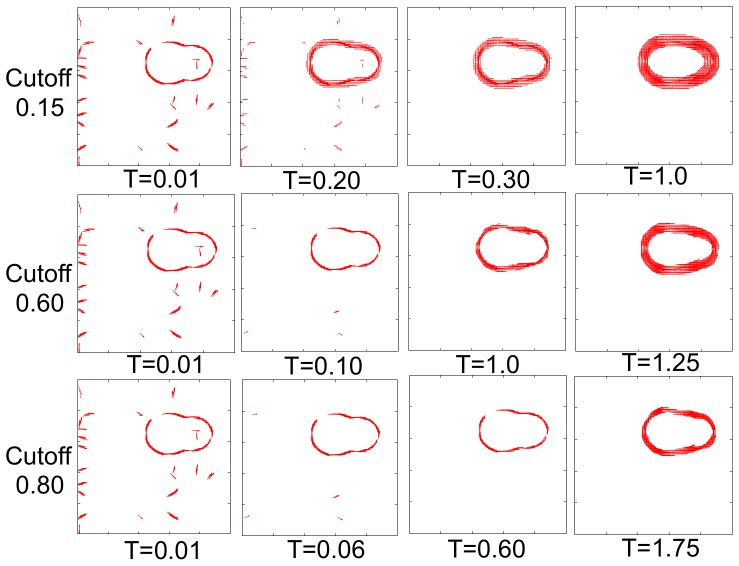
Neural dynamics at different cutoffs. Time evolution of a sample image at different cutoff values. At a lower cutoff the occlusions fills rapidly, but it takes longer to suppress the clutter. At higher cutoffs clutter removes quickly, while it takes longer to fill the gaps. Notice the spurious activity around the contours at longer times. This spurious activity is dominant at lower cutoffs.

We quantified the performance in terms of precision, 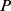, and recall, 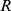. Precision determines the fraction of the total field activity integrated over the image that matches the actual target contour (visible and occluded/invisible). Recall gives the fraction of the target contour that has been recovered. 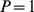 means that there is no clutter, and 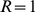 means that all parts of the contour have been identified. For a successful contour detection, we must have 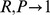 simultaneously. Both 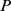 and 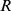 depend on the cutoff used to decide which neurons are considered active (larger cutoff degrades clutter faster, but slows down occlusion filling), and on the time of the simulation (Fig. 5). Hence different cutoffs and times must be explored.

**Figure 6:**
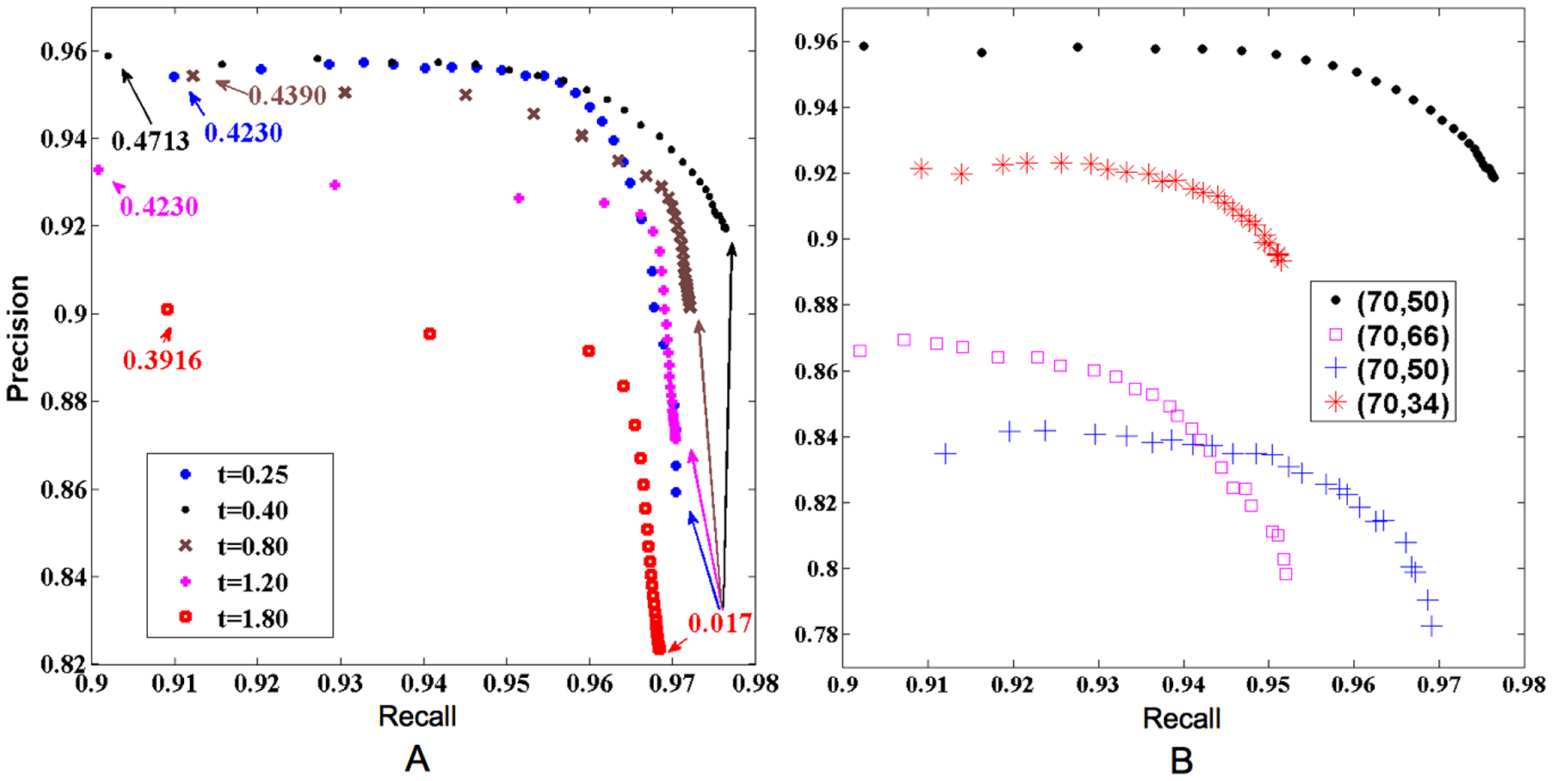
Precision vs Recall with an absolute cutoff. (A) 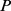 vs 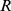 averaged over 500 randomly generated images at various simulation times starting with 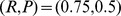. The numbers indicate cutoff values for a specific data point at the corresponding simulation time. Note the weak dependence on the cutoff. The simulation lengths of 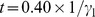 (black dots) produces the curve with the best precision and recall combination. (B) 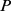 vs 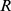 with different starting values of precision and recall averaged over 100 randomly generated images, but with the same model parameters. Legend indicates the initial 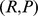. The black dots are the same as in the top panel. Red 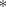
's correspond to a lower initial precision (more clutter), compared to the black dots. Blue 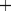
's stand for the same initial 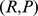 as black, but with the target partitioned into more shorter segments (a larger number of occlusions). Pink 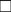
's correspond to higher initial precision (less clutter), but the clutter elements are longer and harder to suppress.

Figure 6 **A** gives the variation of precision and recall at various cutoffs at particular times during the simulation. At 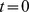, 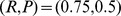 on average, i. e., initially about 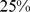 of the target is invisible and the total lengths of the clutter and the target segments are nearly equal. At 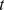 as small as 0.25 (with 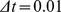), 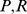 are above 0.9 simultaneously for a large set of cutoff values (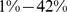). Since we present the stimulus instantaneously only, its effect eventually decreases with time. Thus there is a time that optimizes performance, at which the precision vs. recall curve majorates the same curves for other times. For the data-set in Fig. 6, this optimal time is 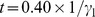 (40 numerical iterations), where the curve reaches 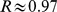 and 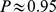 simultaneously.

**Figure 7:**
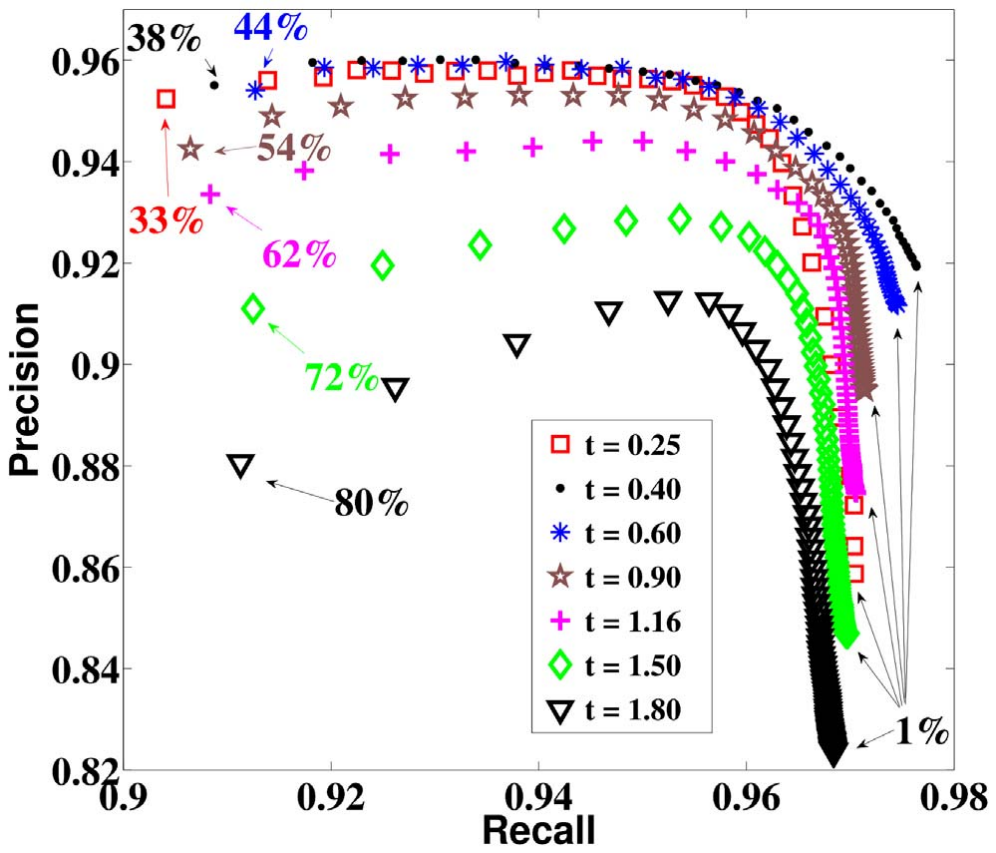
Precision vs Recall with a relative cutoff. 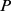
 vs 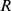 averaged over 500 randomly generated images at various simulation times starting with 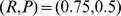. The numbers indicate cutoff values for a specific data point in terms of the percentage of the maximum activity of the field at the corresponding time. Note the similarity with the results in case of absolute cutoff values (Figure 6**A**).

Performance depends only weakly on the *ad hoc* details of the simulations and the data. For example, defining the threshold parameter not as an absolute value, but as a fraction of the maximum activity of the field at a given time point did not change the precision-recall curves much (Fig. 7). Similarly, different amounts of initial clutter had only a moderate effect if the length of the clutter elements remained the same (Fig. 6 **B**). This is because the time scale of the clutter decay depends on the size of the segments, and not on their number. For longer segments, the decay takes longer, and hence the optimal processing time increases. The optimal processing time also increases with the linear dimension of the occlusions present in the target amoebas and with the number of occlusions (Fig. 6 **B**). However, for all of these cases, the maximum precision and recall remain simultaneously high.

## Discussion

We developed a *continuum, coarse-grained model* of V1 to study contour detection in complex images, which is substantially simpler than other models in the literature, and yet still performs nontrivial visual computation. While borrowing heavily from previous research, our model differs from most previous approaches by forgoing individual neurons and describing the neural activity as a parity-symmetric continuous director field, which makes expressions for Hebbian connectivity and solutions of the model dynamics expressible in the closed form. We incorporate some experimentally observed properties of the visual neural dynamics, namely non-linear excitation, thresholding, cross orientation suppression, local relaxation, global suppression, and, crucially, co-circular excitatory connectivity [19], which brings long-range context to local edge detection.

The model identifies long object contours in computer-generated images with simultaneous recall and precision of over 90% for many conditions. It happens even though initially large parts of objects are invisible (potentially lowering recall), and clutter is present (decreasing precision). The model fills in the occlusions and filters out the clutter based on the presence or absence of co-circular contextual edge support. In addition to the substantial simplification, this ability to *fill in the occlusions* particularly distinguishes our approach from the previous work on co-circular excitatory feedback [19], [20]. It remains to be seen to which extent the performance is affected by more natural statistics of images, and by the presence of stochasticity and synaptic plasticity in neural dynamics.

The model performs on par or better than agent-based three-dimensional models (two spatial dimensions and one orientation preference dimension), with complex, empirically specified co-circular interaction kernel [20]. This illustrates that discreteness of neurons, existence of the orientation preference as an independent variable, and intricate details of the kernel are *not crucial* for the studied visual processing function. The reduced complexity is not only conceptually appealing, but also can result in more efficient computational implementations. For example, it should be possible to augment practical feedforward models of object detection, such as [10], with the laterally connected layer developed in this work. We expect this to lead to improvements in object recognition performance.

The model makes predictions that can be tested experimentally, such as regarding the amount of neural excitation in V1 as a function of the computation time and the duration of exposure to an image. Additionally, it predicts that the neural activity localizes to long contours with time, which can be tested with various imaging technologies. Finally, it can be used to predict the dependence of the contour detection performance on the statistical structure of images and on the exposure time. Testing such predictions in psychophysics experiments [20] will be a subject of the future work.

Finally, we notice that the neural field 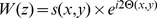 can be mapped exactly onto the Landau - de Gennes order parameter for a two-dimensional nematic liquid crystal
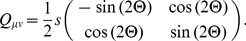
(7)


This may help solve a crucial difficulty in implementing an artificial laterally-interacting neural model: the computational cost of long-range communication. Indeed, one can think of materials with symmetry and dynamical properties such that the neural computation and the communication are performed by the intrinsic dynamics of the material itself. Potential implementations can include polarizable liquid crystals with long-range magnetic interactions, polar colloidal materials, or heterogenous solid state materials with long-range connectivity. The liquid crystal analogy suggests the use of the well-developed repertoire of theoretical physics to understand the impact of different terms in the model neural dynamics, Eq. (1). In particular, one can hope that the future renormalization group treatment of this dynamics will reveal the terms in the interaction kernel 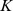 that are relevant for its long-time, long-range aspects.

## References

[1] Felleman D, Van Essen D (1991) Distributed hierarchical processing in the primate cerebral cortex. Cerebral cortex 1: 1. 1822724 10.1093/cercor/1.1.1-a

[2] Creutzfeldt O, Nothdurft H (1978) Representation of complex visual stimuli in the brain. Naturwissenschaften 65: 307. 673010 10.1007/BF00368371

[3] Hubel D, Wiesel T (1968) Receptive fields and functional architecture of monkey striate cortex. J Physiol 195 10.1113/jphysiol.1968.sp008455 PMC1557912 4966457

[4] Hubel D, Wiesel T (1962) Receptive fields, binocular interaction and functional architecture in the cat's visual cortex. J Physiol 160: 106. 14449617 10.1113/jphysiol.1962.sp006837 PMC1359523

[5] Wallis G, Rolls E (1997) Invariant face and object recognition in the visual system. Progr Neurobiol 51: 167. 10.1016/s0301-0082(96)00054-8 9247963

[6] Itti L, Koch C, Niebur E (1998) A model of saliency-based visual attention for rapid scene analysis. Pattern Analysis and Machine Intelligence, IEEE Trans 20: 1254.

[7] LeCun Y, Bottou L, Bengio Y, Haffner P (1998) Gradient-based learning applied to document recognition. Proc IEEE 86: 2278.

[8] Thorpe S, Delorme A, van Rullen R (2001) Spike-based strategies for rapid processing. Neural Netw 14: 715. 11665765 10.1016/s0893-6080(01)00083-1

[9] Boureau YL, Bach F, LeCun Y, Ponce J (2010) Learning mid-level features for recognition. In: Computer Vision and Pattern Recognition (CVPR), 2010 IEEE Conference on. p. 2559.

[10] Serre T, Oliva A, Poggio T (2007) A feedforward architecture accounts for rapid categorization. Proc Natl Acad Sci USA 104: 6424. 17404214 10.1073/pnas.0700622104 PMC1847457

[11] Riesenhuber M, Poggio T (1999) Hierarchical models of object recognition in cortex. Nat Neurosci 2: 1019. 10526343 10.1038/14819

[12] Riesenhuber M, Poggio T (2000) Models of object recognition. Nat Neurosci 3: 1199. 11127838 10.1038/81479

[13] Hinton G, Salakhutdinov R (2006) Reducing the dimensionality of data with neural networks. Science 313: 504–507. 16873662 10.1126/science.1127647

[14] LeCun Y, Huang F, Bottou L (2004) Learning methods for generic object recognition with invariance to pose and lighting. In: Computer Vision and Pattern Recognition, Proc 2004 IEEE Comp Soc Conf. volume 2, p. II.

[15] Stettler D, Das A, Bennett J, Gilbert C (2002) Lateral connectivity and contextual interactions in macaque primary visual cortex. Neuron 36: 739. 12441061 10.1016/s0896-6273(02)01029-2

[16] Angelucci A, Levitt J, Walton E, Hupe J, Bullier J, et al (2002) Circuits for local and global signal integration in primary visual cortex. J Neurosci 22 10.1523/JNEUROSCI.22-19-08633.2002 PMC6757772 12351737

[17] Colonnier M, O′Kusky J (1981) Number of neurons and synapses in the visual cortex of different species. Revue canadienne de biologie/editee par l′Universite de Montreal 40 7244322

[18] Field D, Hayes A, Hess R (1993) Contour integration by the human visual system: Evidence for a local “association field”. Vision Res 33: 173. 8447091 10.1016/0042-6989(93)90156-q

[19] Parent P, Zucker S (1989) Trace inference, curvature consistency, and curve detection. Pattern Analysis and Machine Intelligence, IEEE Trans 11: 823.

[20] Gintautas V, Ham M, Kunsberg B, Barr S, Brumby SP, et al (2011) Model cortical association fields account for the time course and dependence on target complexity of human contour perception. PLoS Comp Biol 7: e1002162. 10.1371/journal.pcbi.1002162 PMC3188484 21998562

[21] Zweck J, Williams LR (2004) Euclidean group invariant computation of stochastic completion fields using shiftable-twistable functions. Journal of Mathematical Imaging and Vision 21: 135–154.

[22] Bressloff PC, Cowan JD (2003) The functional geometry of local and horizontal connections in a model of v1. Journal of Physiology-Paris 97: 221–236. 10.1016/j.jphysparis.2003.09.017 14766143

[23] Wolf F, Geisel T (1998) Spontaneous pinwheel annihilation during visual development. Nature 395: 73. 9738500 10.1038/25736

[24] Wolf F, Geisel T (2003) Universality in visual cortical pattern formation. Journal of Physiology-Paris 97: 253–264. 10.1016/j.jphysparis.2003.09.018 14766145

[25] DeAngelis G, Robson J, Ohzawa I, Freeman R (1992) Organization of suppression in receptive fields of neurons in cat visual cortex. J Neurophysiol 68: 144. 1517820 10.1152/jn.1992.68.1.144

[26] de Gennes P, Prost J, Pelcovits R (1995) The physics of liquid crystals. Phys Today 48: 70.

[27] Gilbert C, Wiesel T (1989) Columnar specificity of intrinsic horizontal and corticocortical connections in cat visual cortex. J Neurosci 9: 2432. 2746337 10.1523/JNEUROSCI.09-07-02432.1989 PMC6569760

[28] Kovacs I, Julesz B (1993) A closed curve is much more than an incomplete one: Effect of closure in figure-ground segmentation. Proc Natl Acad Sci USA 90: 7495. 8356044 10.1073/pnas.90.16.7495 PMC47168

[29] Jeffress LA (1948) A place theory of sound localization. J Comp Physiol Psychol 41: 35. 18904764 10.1037/h0061495

[30] Dayan P, Abbott L (2005) Theoretical Neuroscience. MIT Press

[31] Miconi T, VanRullen R (2010) The gamma slideshow: object-based perceptual cycles in a model of the visual cortex. Front Hum Neurosci 4: 205. 21120147 10.3389/fnhum.2010.00205 PMC2992033

[32] Altmann CF, Bülthoff HH, Kourtzi Z (2003) Perceptual organization of local elements into global shapes in the human visual cortex. Current Biology 13: 342–349. 12593802 10.1016/s0960-9822(03)00052-6

